# Information Extraction Framework for Disability Determination Using a Mental Functioning Use-Case

**DOI:** 10.2196/32245

**Published:** 2022-03-18

**Authors:** Ayah Zirikly, Bart Desmet, Denis Newman-Griffis, Elizabeth E Marfeo, Christine McDonough, Howard Goldman, Leighton Chan

**Affiliations:** 1 Rehabilitation Medicine Department Clinical Center National Institutes of Health Bethesda, MD United States; 2 Whiting School of Engineering Johns Hopkins University Baltimore, MD United States; 3 Malone Center for Engineering in Healthcare Johns Hopkins University Baltimore, MD United States; 4 Department of Biomedical Informatics University of Pittsburgh Pittsburgh, PA United States; 5 Department of Occupational Therapy Tufts University Medford, MA United States; 6 School of Health and Rehabilitation Science University of Pittsburgh Pittsburgh, PA United States; 7 Department of Psychiatry School of Medicine University of Maryland Baltimore, MD United States

**Keywords:** natural language processing, text mining, bioinformatics, health informatics, machine learning, disability, mental health, functioning, NLP, electronic health record, framework, disability, EHR, automation, eHealth, decision support, functional status, whole-person function

## Abstract

Natural language processing (NLP) in health care enables transformation of complex narrative information into high value products such as clinical decision support and adverse event monitoring in real time via the electronic health record (EHR). However, information technologies for mental health have consistently lagged because of the complexity of measuring and modeling mental health and illness. The use of NLP to support management of mental health conditions is a viable topic that has not been explored in depth. This paper provides a framework for the advanced application of NLP methods to identify, extract, and organize information on mental health and functioning to inform the decision-making process applied to assessing mental health. We present a use-case related to work disability, guided by the disability determination process of the US Social Security Administration (SSA). From this perspective, the following questions must be addressed about each problem that leads to a disability benefits claim: *When did the problem occur and how long has it existed? How severe is it? Does it affect the person’s ability to work?* and *What is the source of the evidence about the problem?* Our framework includes 4 dimensions of medical information that are central to assessing disability—temporal sequence and duration, severity, context, and information source. We describe key aspects of each dimension and promising approaches for application in mental functioning. For example, to address temporality, a complete functional timeline must be created with all relevant aspects of functioning such as intermittence, persistence, and recurrence. Severity of mental health symptoms can be successfully identified and extracted on a 4-level ordinal scale from absent to severe. Some NLP work has been reported on the extraction of context for specific cases of wheelchair use in clinical settings. We discuss the links between the task of information source assessment and work on source attribution, coreference resolution, event extraction, and rule-based methods. Gaps were identified in NLP applications that directly applied to the framework and in existing relevant annotated data sets. We highlighted NLP methods with the potential for advanced application in the field of mental functioning. Findings of this work will inform the development of instruments for supporting SSA adjudicators in their disability determination process. The 4 dimensions of medical information may have relevance for a broad array of individuals and organizations responsible for assessing mental health function and ability. Further, our framework with 4 specific dimensions presents significant opportunity for the application of NLP in the realm of mental health and functioning beyond the SSA setting, and it may support the development of robust tools and methods for decision-making related to clinical care, program implementation, and other outcomes.

## Introduction

Over the past 2 decades, the use of data-driven informatics techniques to aid in clinical decision-making has increased across the fields of computer science, bioinformatics, and medicine [1]. Natural language processing (NLP), which enables analysis of complex information recorded in narrative text format, has been a key driver of informatics successes in health care. Applications such as automated report analysis for clinical decision support and adverse event monitoring in the electronic health record (EHR) have been widely adopted [2-5]. However, informatics technologies for mental health have consistently lagged because of the complexity of measuring and modeling mental health and illness. The expansion of medical NLP technologies from clinical applications into the realm of complex administrative tasks such as claims evaluations and benefits administration [6,7] has highlighted the need for improved tools for analyzing information on mental health and function, which play a significant role in key health outcomes such as disability [8].

One of the primary goals of NLP in health data is to analyze narrative medical texts such as medical histories, physical examinations, and standardized assessments to extract the data needed to inform decision-making processes. The use of NLP to support these goals for management of mental health conditions has not yet been explored in depth. Our research group develops NLP models to support the information needs of the US Social Security Administration (SSA) disability determination process. Through its disability programs—Social Security Disability Insurance (SSDI) and Supplemental Security Income (SSI)—the SSA is the largest federal provider of financial assistance to workers with disabilities and their families. Because of the impact of functional abilities on both the individuals with disabilities and the society, it is essential that a person’s functional abilities are characterized both comprehensively and efficiently in the disability determination process. Multiple sources of information are used to understand a person’s ability to work. Given the complexity of the disability determination process, there is interest in developing approaches that enhance the validity of and confidence in the information across sources. While our work is motivated by the SSA’s focused use-case, the SSA setting reflects fundamental challenges in the development and broad application of medical informatics technologies. The SSA leverages data from all types of health care providers and EHR systems across the United States. Therefore, informatics tools must be robust to significant heterogeneity in documentation—a known challenge for medical NLP research [9]. The volume of applications for disability benefits that the SSA must process is also extraordinarily high—over 2 million applications every year since 2004 [10]—and informatics tools must therefore support rapid processing of high-volume data. Finally, and a key motivating factor for our work, the SSA’s decision-making processes must incorporate diverse health and function information from all domains of human experience. The SSA setting thus provides an invaluable environment for learning how to translate the potential of NLP tools into practical, reliable tools for real-world applications.

### Contributions of This Paper

In this paper, we propose a framework for the advanced application of NLP methods to identify, extract, and organize functioning information to inform the decision-making process applied to assessing functioning and disability. While the framework is applicable to mental and physical functioning use cases alike, this paper focuses on mental functioning. We found no literature that directly addresses our decision-support use-case for mental health and function; therefore, we developed a conceptual framework for synthesizing prior NLP research to create decision support tools for use in assessing SSA’s definition of disability. Our framework includes 4 dimensions of medical information that are central to assessing disability—temporal sequence and duration, context, severity, and information source. Findings of this work are intended to inform the development of instruments that will support the decisions of disability adjudicators in the SSA’s stepwise process of disability determination and have implications for a broad array of individuals and organizations responsible for assessing mental health function and ability. Further, our framework presents significant opportunity for the application of NLP in the realm of mental and physical health and functioning beyond the SSA setting, and it can support the development of robust tools and methods for decision-making related to clinical care, program implementation, and other outcomes.

### Background

The US SSA administers the largest federal assistance programs in the United States, including 2 disability programs: SSDI and SSI. Both programs are based on a statutory definition of disability as the inability to engage in any substantial gainful activity by reason of any medically determinable physical or mental impairment(s), which can be expected to result in death or which has lasted or can be expected to last for a continuous period of not less than 12 months. The SSA’s disability determination process is a stepwise process for evaluating individuals according to criteria that operationalize the statutory definition of disability. The process is based on federal regulatory standards that include both financial and medical criteria. Applicants are either allowed or denied at each step or move on to further evaluation in the subsequent steps. The process is administered by state Disability Determination Service agencies. In step 1, applicants are denied if they work and earn more than the threshold for substantial gainful employment. In step 2, applicants are screened based on whether medical evidence supports the existence of a severe impairment. In step 3, the applicant’s medical evidence is compared to codified clinical criteria for various medical impairments, called the Listing of Impairments (Listings). When impairments “meet” or “equal” the Listings, the applicants are allowed. Applicants who are not allowed at step 3 move on to steps 4 and 5, which assess vocational factors such as the “residual functional capacity” of the individual as well as the applicant’s age, education, and relevant work experience. In step 4, adjudicators within the Disability Determination Service assess whether the applicant can work in any of their past jobs. If the adjudicator determines that an applicant can work in a previous job, the applicant is denied. Otherwise, in step 5, the Disability Determination Service adjudicators evaluate whether the applicant can perform any work in the national economy. There has been internal effort at the SSA to improve accuracy and timeliness of the disability adjudication process, and external groups have been engaged to assist with this. Expert panels and evaluations of the processes have resulted in recommendations for more systematic integration of functional information into adjudication decisions [11,12].

As part of the adjudication process, adjudicators amass a body of evidence referred to as the Medical Evidence of Record (MER), composed of information collected from multiple sources to characterize a person’s potential ability to work. The MER forms the primary resource from which it is determined if an individual meets the SSA’s statutory definition of disability. Therefore, the adjudicator must extract a variety of information from the MER, including medical evidence, medical opinion, and lay evidence, to support a decision on disability under this statutory definition.

A primary challenge for accuracy in the disability determination process is that adjudicators must access *all* relevant information from the MER for their decision, including information about both health conditions and functional abilities that relate to work. MER for a single individual may include dozens to hundreds of clinical reports, which imposes a significant burden on the adjudicator to rapidly process extensive medical evidence. Automated analysis of these documents with NLP thus has significant potential to assist adjudicators in the evidence review process and to support efficiency of the process. Our research group has developed novel NLP technologies for automated identification of functional status information in medical evidence [7], thus providing high-coverage retrieval of information related to mobility limitations [13-15] and categorization of this evidence according to the World Health Organization’s International Classification of Functioning, Disability and Health [16]. Expansion of these technologies to mental health and function requires adaptation to the conceptual frameworks that characterize mental function, as outlined in the sections that follow.

For the purposes of this paper, we focus on mental health functioning, that is ways in which a person’s underlying cognition, emotions, and behaviors affect their ability to perform daily activities including work tasks and participation, for example, a person’s ability to regulate their emotions in stressful situations, multitask, or solve problems. This operationalization is based on the biopsychosocial model of health and function by the World Health Organization’s International Classification of Functioning, Disability and Health. In this model, disability results from a gap between a person’s underlying ability and the context in which they are performing various activities (eg, work participation [17,18]). This model highlights the fact that diagnostic information is necessary but not sufficient to understand a person’s ability to participate in meaningful activities such as employment. In clinical contexts, information on functioning is critical to understanding the impact of conditions on people in their personal and environmental contexts and to develop an effective management plan. Recent work has demonstrated initial feasibility of applying NLP methods to mental health-related topics, including psychiatric readmission and symptoms of severe mental illness (SMI), as well as to mental health and suicide risk within nonclinical texts [19-23]. There is little evidence of the potential for NLP methods to characterize functional and behavioral manifestations of mental health in a person’s daily life.

### Information Needs for Analyzing Mental Health and Functioning

For disability adjudication, a wide array of information is needed about the following specific areas of mental functioning that a person uses in a work setting: understanding, remembering, or applying information; interacting with others; concentrating, persisting, or maintaining pace; and adapting and managing oneself. Evidence in the MER may reflect a physical or mental *impairment* that may affect these areas of functioning, an observed *limitation* in one of these areas of functioning, or both. The adjudicator’s task is therefore to evaluate the level of severity or degree to which the medically determinable mental impairment affects the 4 areas of mental functioning (ie, limitations) and an individual’s ability to function independently, appropriately, effectively, and on a sustained basis.

Thus, adjudicators must organize and synthesize the medical evidence in the following 4 distinct dimensions to understand the trajectory of mental function in an individual and its impact on work capacity: temporal information including sequence and duration, severity of extracted mentions of functioning, the context with respect to work and work-related information, and the source of the information.

**Figure 1:**
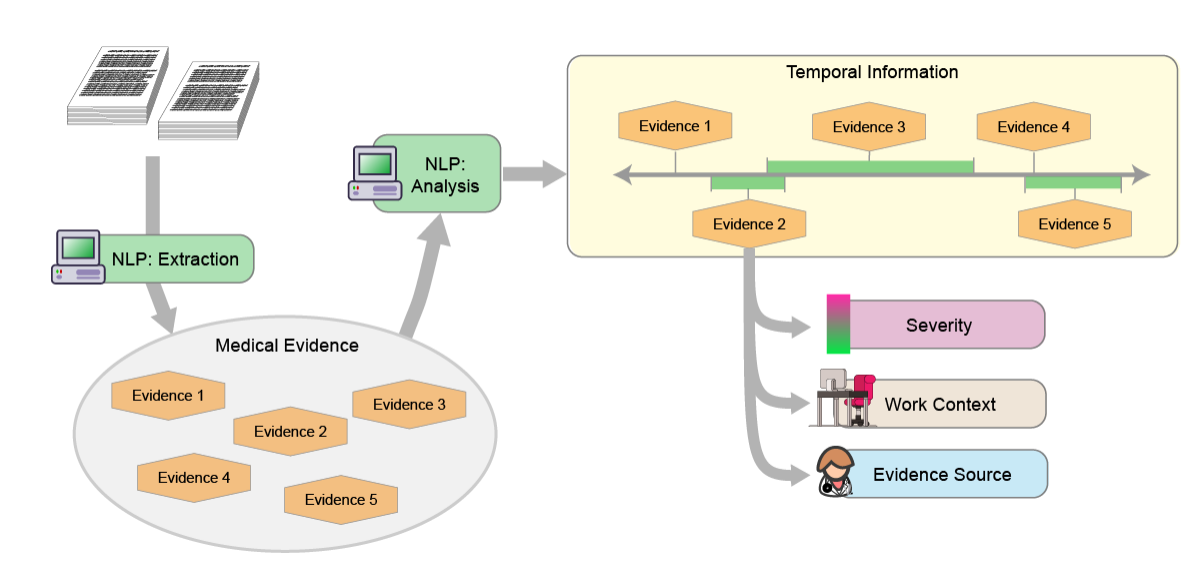
Conceptual pipeline for analysis of information on function in medical records. NLP: natural language processing.

We envision the use of NLP technologies to transform raw evidence found in medical records (MER in the SSA context) into a structured presentation of evidence illustrating each of these 4 factors to SSA adjudicators. Figure 1 illustrates the conceptual structure of such an analytic pipeline. Evidence is first extracted from the MER documents and then ordered into a temporal sequence, with each piece of evidence annotated with the severity of impact on function, the relevant work context, and the source of the evidence.

The remainder of this paper describes the existing NLP literature related to these 4 tasks and highlights how each can be addressed in the area of mental health and function.

## Methods

### Literature Search

We conducted a scoping review of NLP approaches, models, and methods to characterize functional status in free text in the biomedical, clinical, mental health, and disability domains from 1994 to 2021. We searched Google Scholar, which indexes not only PubMed but also conferences and workshops that may be relevant to our scope of interest such as the Association for Computational Linguistics (ACL) conferences, BioNLP, Clinical NLP, and CLPsych. Our search yielded a small number of publications in special workshops, such as AI4Function, that discuss the extraction of physical function information (eg, mobility) but do not address mental health function. We did not find any articles in our area of focus. To expand our search, we used keywords in Google Scholar related to the 4 dimensions of our proposed framework to find articles that describe approaches relevant to each dimension but an area different from functioning. Examples of keywords used include “temporal ordering,” “clinical temporal ordering,” “event extraction,” “NLP and mental health,” “symptom severity,” “environmental context,” “personal context,” and “author attribution.”

### Findings From Existing Work

Disability in the SSA context is defined as the inability to engage in substantial gainful activity for at least 12 months because of a physical or mental impairment and is assessed both in terms of the *trajectory* of a person’s function and the *context* of how it relates to work. As the disability adjudication process also involves collecting MER data from a variety of providers, it is critical to understand how different pieces of evidence relate to one another in terms of the perspective of the information’s *source* (eg, the disability claimant, a medical professional with an established relationship with the claimant, an outside consultant). Thus, for each piece of evidence in the MER, an adjudicator must be able to answer the following 4 questions: *When and for how long was an impairment or associated limitation true? How severe or intense is the impairment or limitation? Does the impairment or limitation affect work?* and *Who reported the impairment or limitation and how convincing is it as evidence?*

In this paper, we present an overview of relevant NLP research and methodologies that can help the adjudicator extract relevant information for these 4 questions. However, it is important to note that building solutions to address these 4 questions requires the ability to identify mental impairments either manually or via automated algorithms. In this paper, we choose to focus on addressing the 4 dimensions or questions only and assume that information on mental impairment is available. We justify our choice by the following factors: the availability of extraction systems that, given annotated data, can extract mentions of mental health impairments with high confidence in EHRs [24] and clinical text [21] and can extract these mentions using available International Classification of Diseases codes; and the novelty and urgency of the proposed 4 dimensions and the lack of available studies to address them. The mentions include observations such as “The patient was not able to concentrate on the given tasks for more than 5 minutes during the exam.”

### Temporal Information

Temporal information includes duration and temporal sequencing. In our use-case, the SSA’s statutory definition of disability requires specific definition and sequential information. The disability is due to a mental impairment, so the impairment must precede the functional limitation.

Temporal sequencing or temporal reasoning has been an active research area in NLP and data mining for a long time. Its importance comes from its applicability to many tasks such as summarization [25], question answering [26], and medical informatics [27]. Given the similarity of the medical utilization task to our use-case, we will mainly focus on reviewing NLP techniques developed in that area and how we can integrate them into our framework.

Temporal reasoning usually includes the following aspects: identifying the targeted events for the task (eg, treatments, diagnosis, symptoms, or medications); and defining time in a machine-readable way that is relevant to the domain and task and extracting temporal information related to the targeted events (eg, in a medical informatics setting, we care about the duration of symptoms or frequencies of medications) [28].

In the context of mental health functioning, the events of interest are events related to mental health conditions and impairment, which can be separated into the following 3 distinct categories: *persistent*, the impairment continues to exist over a prolonged time without interruptions of some criterion duration; *intermittent*, the impairment occurs at irregular intervals and is observed in a temporal sequence with interruptions greater than the criterion duration that defines persistent; and *recurring*, the impairment occurs periodically or repeatedly. Although we can think of recurring as a special case of intermittent, a recurrent mental functioning event is observed again after a period of some specified duration that is longer than the minimum duration defining intermittent.

While this list mainly focuses on the disability use-case, it presents a framework for researchers to structure their problem using all or a subset of our temporal formalization based on the targeted use-case, task, and domain. Our suggested framework differs from other NLP techniques for temporal sequencing because we need to consider nuances that accompany the mention of temporal information. For instance, a sentence such as “The patient reports having lack of interest mainly during the morning hours when it is the weekend,” suggests the need for a system that can highlight the time: weekend morning hours, associated with lack of interest.

Although we introduce a slightly different framework for temporal sequencing, existing NLP methods can be applied to mental health functioning, especially given that most time expressions in medical notes are in the format of date and frequency (eg, how many times per week/day). For instance, temporal recognition and reasoning have played a significant role in information extraction systems [28-30]. Denny et al [29] developed a system that identifies the temporal information and status of colonoscopy events within EHRs with high precision and recall (>.9). In the area of mental health, Viani et al [28] focused on temporal expression extraction to help estimate the duration of untreated psychosis. The temporal information extraction helps in identifying in EHRs when the psychosis symptoms started (onset) and when the treatment was first initiated. Examples of temporal expressions from the paper include mentions such as “started hearing voices at the age of 16, these hallucinations were not elicited during today’s exam.” This is highly relevant to our use cases and to identify the 3 temporal formalizations of persistent, intermittent, and recurring.

To build NLP systems that can identify temporal information, the availability of annotated data sets with temporal information is critical. Although such data sets outside the clinical and medical domains have been publicly available and more easily accessible, such as the ACE 2005 Multilingual Training Corpus [31], the clinical domain imposes more limitations, especially mental health, given the sensitivity of this information and privacy concerns. Examples of annotated data sets for temporal reasoning in clinical text are THYME corpus [32], where 1254 deidentified oncology notes from the Mayo Clinic have been annotated using the ISO-TimeML specification [33]. Sun et al [34] introduced one of the most popular data sets for temporal reasoning in their i2b2 data set that contains 310 discharge summaries. In both these data sets, the focus is on 4 time annotation categories: date, includes both actual dates in addition to mentions such as yesterday and tomorrow and duration, frequency, and time (eg, 3 PM, in the afternoon).

In another data genre, but within the area of mental health, there have been efforts to introduce temporally annotated data sets such as RSDD-Time [35]. This data set is extracted from social media posts that focus on self-reported patients who are diagnosed as having depression. The annotation includes temporal information relevant to when the diagnosis occurred and if the condition is still present.

Given our use-case, we believe that the i2b2 annotation scheme would serve our goals for identifying when the impairment or symptoms occurred and determining for how long the symptoms or impairments lasted.

With regard to methods, researchers used a variety of machine learning techniques such as logistic regression that is especially effective for a small training sample size of less than 500 [36]. Recent advances in the contextualized embeddings [37,38] improved the performance of NLP tasks, including temporal ordering in the clinical domain [39-41]. For instance, Med-BERT [42], a language model that is trained and fine-tuned on the EHR data set, yields a performance that is comparable to that of deep learning techniques on data sets that are almost 10 times larger.

### Severity

The severity of a symptom or functional limitation is an important factor for psychological assessments and psychometric benchmarks, where it is often recorded using a 4-level ordinal scale or as a score that is discretized into that scale. A typical scale includes absent, mild, moderate, and severe labels [43]. The latter 3 labels are frequently employed for the disorders described in the Diagnostic and Statistical Manual of Mental Disorders Text Revision Fourth Edition (DSM-IV-TR), which permit severity specifiers. An advantage of this scale is that mental health clinicians and laypeople alike readily understand it, and it has been adopted in computational approaches for severity classification as well.

Filannino et al [44] describe an NLP shared task focused on symptom severity prediction in neuropsychiatric evaluation records with an exclusive focus on positive valence events, objects, or situations that are harmful but attractive to patients to the point that they are actively engaged despite the consequences. Positive valence is classified on the aforementioned 4-level scale at the patient level and assesses lifetime maximum severity. As such, this task differs from our approach in that it is not time dependent or resolved at the individual mention level. Filannino et al [44] report that in this relaxed use-case, the task can be accomplished automatically with close to human performance.

Severity classification has also been actively researched in suicide risk assessment for patients and individuals on social media. For instance, Shing et al [45] and Zirikly et al [46] introduce an annotated Reddit data set for users with and without depression, each of whom received a suicide risk assessment score on a 4-level scale (none, low, moderate, high). Zirikly et al [46] organized a shared task for advanced automatic user suicide risk classification and provided baseline systems using deep learning models (eg, convolutional neural network) and machine learning models that require feature engineering. Examples of features that are commonly used in NLP methods for the mental health domain and emotion detection and classification are n-grams, lexicons such as the Linguistic Inquiry and Word Count [47], and emotion-word dictionaries [48], topic models, and Reddit usage metafeatures. Top-ranked systems could distinguish between low-risk and high-risk users, but fine-grained 4-level scale classification results indicate the need for further research.

Jackson et al [21] introduced an annotated data set for SMI using clinical text from the Clinical Record Interactive Search system in a cohort of 18,761 patients with SMI and 57,999 individuals without SMI. The authors used a support vector machine model to extract symptoms associated with SMI from discharge summaries. While the data and model for this task are relevant for the severity classification use-case, it does not address severity classification directly.

We can conclude that no severity classification models currently exist for mental health signs and symptoms, but there is a growing body of work on severity classification at the patient level. For clinical symptoms more broadly, Koleck et al [49] performed a systematic review of NLP approaches for processing symptoms in free-text EHR narratives. They found that out of 14 studies, the large majority used documentation occurrence or frequency of occurrence to investigate symptoms, and symptom severity was explicitly evaluated in only 1 study: Heintzelman et al [50] used NLP on oncology provider encounter notes to classify the severity of cancer patients’ pain symptoms into no, some, controlled, or severe pain. Koleck et al [49] report accurate extraction of symptom severity with location and duration as important directions for future work on EHR NLP algorithms.

From our literature review, we find that for both mental and physical health, there is ample opportunity for novel work on severity classification of symptoms and functioning and for continued efforts at the patient level.

### Context

The context in which a functional impairment or limitation is experienced or observed is critical to understanding its impact on work-related activities. Functional activity is an outcome of the interaction between an individual (including physical or cognitive impairments in addition to personal identities and preferences) and their physical, social, and cultural environment [51]. Characterization of this multidimensional relationship between environment, personal factors, and functioning is thus highly complex, as reflected by the wide variety of strategies used to capture contextual information in functional measurement [52]. Two themes have emerged in prior literature that make a useful distinction between different types of contextual factors: *social context* (ie, social determinants of health), broader characteristics of an individual’s social situation such as socioeconomic status, education, zip code, and race and ethnicity identifiers, which inform available resources and opportunities for activity [53]; and *individual context*, factors that are more specific to an individual’s activity performance, such as the physical environment for an activity, social roles such as work requirements, and personal preferences such as transportation access or personal values.

While research on social determinants of health has grown rapidly [54-57] due in part to their strong correlation with population-level health outcomes [58], research on individual context and environment—which more directly impacts functioning [59,60]—remains a significant challenge. Conceptual frameworks of disability have grown to recognize the role of both environmental factors and personal factors in functional outcomes, as seen in the World Health Organization’s International Classification of Functioning, Disability and Health. Measures have been developed to characterize environmental factors of function, including physical [61] and psychosocial environment [62]. Such measures can be highly informative regarding functional outcomes [63]. However, they are not systematically used in clinical contexts [64,65] and some work-related aspects of environment remain underspecified even in conceptual models [66]. Functional assessment measures, on the other hand, frequently either control for environment (as in standardized performance measures) or embed environmental characteristics directly into the measurement of function [67,68] rather than capturing them as related variables. In either case, the details of a person’s environment and its role in their functional outcomes are difficult to extract reliably.

Environmental factors are only one part of the contexts in which people function and must be combined with information on personal factors affecting functional outcomes. Two recent studies have developed steps toward systematically capturing personal values and capabilities to inform rehabilitative care for older adults, though automated analysis of this information remains a future direction [69,70]. Individual context is a largely unexplored area for NLP research due in part to the novelty of human functioning as an area of NLP application [71]. In an initial foray, Agaronnik et al [72] used NLP to capture wheelchair usage—which, as an assistive device, may be considered a contextual factor affecting functional outcomes—from clinical data and demonstrated clear utility of this information over structured billing and diagnosis codes alone. More broadly, the flexibility of free text and the availability of NLP tools to analyze it offers greater freedom for recording and analyzing information on salient contextual factors when the full power of more robust but burdensome environmental measures is not needed. We therefore highlight individual context, where social context meets individual activity, as a key direction for future NLP research to enable mental health and function analysis.

### Source Attribution

In the context of the SSA, disability claims can include the following sources of information (Code of Federal Regulations, SSA): objective medical evidence, medical opinions, and lay evidence.

Objective medical evidence includes signs and laboratory tests reported by recognized medical sources. It is characterized by being quantifiable and discernable. This is highly important and indicative of the intensity and persistence of the symptoms and their impact (eg, how pain severity can affect work ability). Medical opinions include relevant information received from both medical and nonmedical sources. Examples of such information are daily activity and other factors relevant to functional limitations caused by pain or symptoms; location, duration, frequency, and intensity of pain or symptoms; and treatment or any other medication used to alleviate the pain or symptoms. Lay evidence consists of information outside of objective medial evidence or medical opinions—assessments of disability or functioning limitation provided by knowledgeable nonmedical sources such as family, teachers, social services personnel, and employers. This will complement the information provided in objective medical evidence and medical opinions to better understand the impairments from multiple perspectives. Moreover, lay evidence is very insightful and important when medical evidence does not provide enough evidence for symptoms [73]. As we note, these types of evidence carry different levels of authority and support for the symptoms of the patient. Therefore, the adjudicators need to evaluate and address each evidence separately given its source to make a more comprehensive decision for disability eligibility. In this section, we will start with an overview of related work in NLP, followed by our recommendations to customize these efforts or build on them to address the needs to source attribution within our proposed framework.

Source attribution, as proposed, correlates with multiple similar tasks in NLP. We will start with an example to showcase options for NLP techniques we can adopt from: *The patient lacks interest in doing anything, his mom mentioned. When the doctor asked the patient, he claimed that he goes to work most of the time. At the end of the visit, the doctor diagnosed the patient with depression based on multiple assessments*. First, as we mentioned previously, we are assuming the availability of an extraction system that can identify and recognize mental health functioning and diagnoses statements. Table 1 depicts the mention, its source, and type of evidence.

**Table 1:** Examples of different mental health functioning.

Mention	Source	Type
The patient lacks interest in doing anything	Mother	Lay evidence (symptom)
He claimed that he goes to work most of the time	Patient	Medical opinion (daily activity)
The doctor diagnosed the patient with depression based on multiple assessments	Doctor	Objective medical evidence (diagnosis)

Identifying who made the statements is similar to the task of identifying author attribution in a dialogue or quoted speech [74]. Although regular expressions can capture simple cases of source attribution of impairments, such as “The patient said,” Pareti et al [75] and O’Keefe et al [76] discussed more advanced techniques for quotes—direct and indirect—attribution in opinion mining. Although they show promising results, these methods have been geared and tested on newswire data. All these techniques require clean and well-structured data, an assumption that is hard to meet, especially given the noise presented in clinical notes [77].

There are some cases in which the doctor or medical expert omits mentioning the source in the note, especially when the observation is generated from them or another medical expert. In such cases, inferring source is difficult as it is not explicitly known or inferred (using coreference resolution). For these scenarios, we suggest that techniques be adopted from author attribution task. This task focuses on identifying the author of a text. This task has been well studied in multiple applications; the most traditional one is assigning anonymous literary to authors [78,79]. Additionally, it has been used in forensics to identify authors that are involved in internet-based activity in different text genres such as online messaging (eg, emails) [80], news text data set [81], and social media [82,83]. However, in our work, we focus on attribution of short text or sentences in notes.

Furthermore, we believe that it would be beneficial to adopt techniques from the intersection of author attribution and coreference resolution [84,85]. We see similarities with event extraction, where we focus on event attributes, mainly participants, when the event describes a mental health functioning mention. Techniques to extract multiple accounts from a narrative, such as the ones described by Zhang et al [86], can be adopted in our work to identify who made the observation or statement.

It is important to note that the attribution problem, as we propose it, requires systems that can identify mental health functioning mentions (eg, depression, lack of interest). For that goal, availability of annotated data to train and test machine learning systems is essential [87]. Although we earlier addressed the need for annotated data sets that have labels for mental health functioning, we need additional labels for the source attribution problem. The labels need to address who the source is and the type of evidence. However, it is worth pointing out that data sets that have labels solely on the level of evidence are sufficient for our targeted use-case. Labels can be assigned from the 3 types of evidence we mentioned above.

## Discussion

### Applications Beyond Disability Adjudication

We have presented a framework to support extraction of functional information in mental health, which includes 4 main dimensions. There were no existing NLP applications that are directly applied to the characterization of temporality, severity, source, and context. However, we identified relevant work in mental health and other areas that could be used for the advanced application of NLP in the field. Temporal expression extraction and a relevant annotation scheme for identifying onset and duration were presented. A model for extracting severity of functional limitations was presented based on existing ordinal symptom severity ratings. An example of extraction of context was provided based on specific cases of wheelchair use in clinical settings. Finally, alternatives for source attribution were identified among existing approaches.

While our framework is tailored to the SSA disability benefits adjudication process, it has implications for a wide variety of applications outside the SSA context. For example, this approach could be used when extracting information for use in the medical case review process. This process requires expert review of patients’ care history based on medical records to ensure that the treatment provided meets Medical Necessity Criteria. Additionally, this framework may be useful for other review activities, including informing the process for assessing eligibility determinations, individualized education programs, and educational placements for children under the Individuals with Disabilities Education Act. For this paper, we briefly highlight applications for mental health informatics research, functional assessment and program management in the health care setting, and consultations for case-based recommendations in treatment and managed care.

There is significant untapped potential for informatics technologies focusing on mental health and functioning, as evidenced by the interest of the mental health informatics research community in recent years. The use of informatics technologies has grown for the detection and diagnosis of mental health conditions [88], and the use as tools in mental health care delivery is beginning to be explored [89]. Our framework can inform the expansion of these technologies into a longer-term view of the trajectory of mental health and functioning in a person, thus improving the power of predictive analytics and presentation of health information to providers. Murnane et al [90] describe several technological needs for long-term mental health management, including incorporation of social contexts—a key component of our framework for NLP. Rigby et al [91] identified several aspects of mental health care that are still challenging for mental health informatics 2 decades later, including the importance of a longitudinal view. By identifying clear links to existing NLP research, our framework can serve to guide translational NLP research [92] in the mental health domain. This work can help identify both processes for translating existing NLP technologies into robust solutions for application in mental health research and care as well as new research questions for progress on the needs of mental health informatics.

There are numerous potential specific applications of our approach to using NLP for extraction of information from various sources to assist with the assessment of mental functioning. These are applications that require a review of medical and health-related information to assess functioning for the support of various clinical and other human service processes. The most common application would be reviews of clinical records to decide on a diagnosis or a course of treatment. A similar approach might be used by a managed care organization to determine the medical necessity of an episode of care or receipt of service. A consultant who is involved in the second opinion on a diagnosis or treatment plan could benefit from a decision-support tool that extracts all the information in a medical record that is relevant to mental functioning. Outside of health care settings, educational organizations and child welfare organizations might use such a clinical review to assess a student’s need for special assistance or accommodation based on impairment in mental functioning. The development of an Individual Education Plan or a 504 plan [93] could use an NLP support tool to extract information from school and medical records to assess the need for special supports.

It is worth noting that this framework and its 4 key elements can be used for and generalized to any area of functioning within the SSA disability program and its statutory definition of work disability.

### Support Tools for Disability Adjudication Need High Sensitivity

Current tools available to extract data related to mental health and function lack the level of sensitivity with respect to the elements in the MER on many types of mental functioning due to a mental impairment. While adjudicators ultimately need information on more fine-grained aspects of temporal sequencing using constructs such as intermittence, persistence, and recurrence, the main challenge is to create a complete timeline with all relevant aspects of functioning. Human decision makers assess the more fine-grained aspects of these characteristics of functioning. NLP systems thus need to extract the information without necessarily making fine-grained distinctions. One needs to know all the fine-grained elements to extract all relevant information even if the NLP tool does not need to make the distinctions. What is true for the granularity of temporality is also true for context, severity, and source, but most importantly, an NLP tool needs to be sensitive so that no information in the MER is overlooked.

## Limitations and Challenges With Respect to NLP for Mental Health Function

Although the domain of mental health in general is attracting more NLP research, these studies focus on classification tasks in terms of diagnosis or identification of high-risk individuals and do not address how these impairments affect the patient’s functioning in both personal and work environments. Thus, there will be challenges and obstacles as research evolves in this domain.

As we described in the paper, the domain of mental health in general and especially mental health functioning is ambiguous and highly semantic. This yields to different interpretations and inconsistencies in annotating documents with mental health functioning mentions and attributes, as consensus by humans is harder to attain. Furthermore, the lack of gold standard and manually annotated corpora for mental health functioning that are essential to build robust extraction solutions highlights the need for the interested community to invest resources in building such corpora to further improve the performance of these solutions.

Although precision, specificity, and sensitivity (ie, recall) metrics are important, we believe that the interested entities, such as the SSA, can directly benefit from tools that focus on sensitivity (ie, higher recall) rather than higher precision and specificity. Such premise extends the invitation for research in categorization and relevance ranking to compensate for the low specificity and precision of such systems. Although we are aware of the importance of that line of research, this paper leaves this work for the future.

## Conclusions and Future Vision

There is tremendous opportunity for the development and application of NLP tools and methods for the characterization of mental functioning. Although we found no literature that directly applied to the 4 main dimensions in the proposed framework, relevant tools and methods were identified. Research and development leveraging this existing work to tailor approaches for the extraction of temporality, severity, source, and context will yield substantial value to the use-case of disability determination and beyond. Future work should focus on developing relevant annotated data sets and tools trained on the key aspects of the 4 mental functioning domains.

## Abbreviations

ACL: Association for Computational Linguistics
DSM-IV-TR: Diagnostic and Statistical Manual of Mental Disorders Text Revision Fourth Edition
EHR: electronic health record
MER: Medical Evidence of Record
NLP: natural language processing
SMI: severe mental illness
SSA: Social Security Administration
SSDI: Social Security Disability Insurance
SSI: Supplemental Security Income
